# The hematopoietic factor GM-CSF (Granulocyte-macrophage colony-stimulating factor) promotes neuronal differentiation of adult neural stem cells in vitro

**DOI:** 10.1186/1471-2202-8-88

**Published:** 2007-10-22

**Authors:** Carola Krüger, Rico Laage, Claudia Pitzer, Wolf-Rüdiger Schäbitz, Armin Schneider

**Affiliations:** 1 Sygnis Bioscience, Im Neuenheimer Feld 515, 69120 Heidelberg, Germany; 2 Department of Neurology, University of Münster, Albert-Schweitzer-Str. 33, 48149 Münster, Germany

## Abstract

**Background:**

Granulocyte-macrophage colony stimulating factor (GM-CSF) is a hematopoietic growth factor involved in the generation of granulocytes, macrophages, and dendritic cells from hematopoietic progenitor cells. We have recently demonstrated that GM-CSF has anti-apoptotic functions on neurons, and is neuroprotective in animal stroke models.

**Results:**

The GM-CSF receptor α is expressed on adult neural stem cells in the rodent brain, and in culture. Addition of GM-CSF to NSCs in vitro increased neuronal differentiation in a dose-dependent manner as determined by quantitative PCR, reporter gene assays, and FACS analysis.

**Conclusion:**

Similar to the hematopoietic factor Granulocyte-colony stimulating factor (G-CSF), GM-CSF stimulates neuronal differentiation of adult NSCs. These data highlight the astonishingly similar functions of major hematopoietic factors in the brain, and raise the clinical attractiveness of GM-CSF as a novel drug for neurological disorders.

## Background

Granulocyte-macrophage colony stimulating factor (GM-CSF) is a hematopoietic growth factor involved in the generation of granulocytes, macrophages, and dendritic cells from hematopoietic progenitor cells [1]. The GM-CSF receptor is a heterodimer of the GM-CSF receptor α subunit and the β subunit which is not directly involved in binding GM-CSF. GM-CSF is used clinically in the field of oncology and hematology [2]. Recently we have identified GM-CSF as a neuronal growth factor in the brain which counteracts apoptosis, and reduces infarct size in stroke models in vivo [3]. GM-CSF has also been identified as a factor involved in arteriogenesis after brain ischemia [4,5]. GM-CSF is therefore the third hematopoietic factor after EPO and G-CSF that has functions in the brain.

GM-CSF has very similar characteristics to G-CSF in the brain in terms of patterns of expression, anti-apoptotic functions, and neuroprotective effects [3]. G-CSF has a prominent effect on the neuronal differentiation of adult neural stem cells [6]. We therefore determined whether GM-CSF might have a similar role also on the differentiation of stem cells.

## Results

### The GM-CSF receptor alpha is expressed on adult neural stem cells

**Figure 1:**
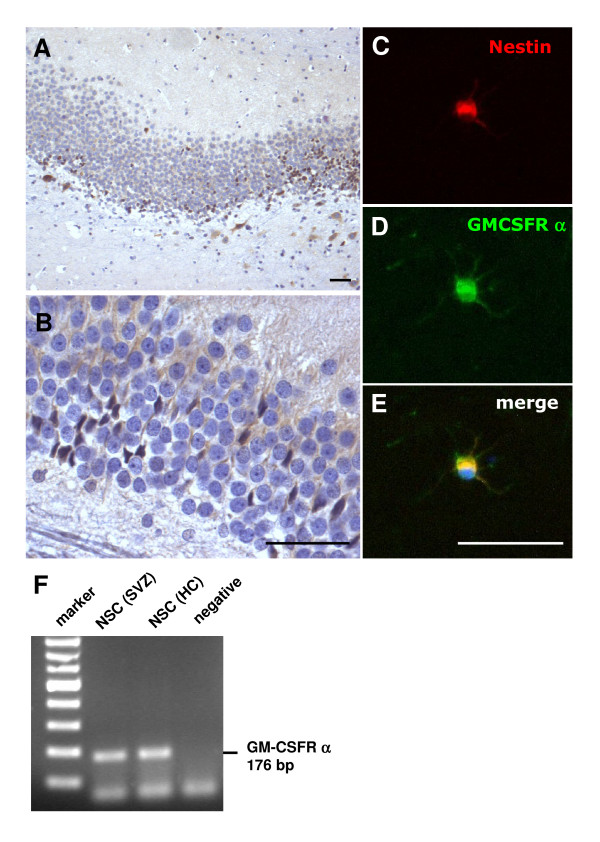
**GM-CSFR alpha is present on adult neural stem cells**. **A, B**, GM-CSF receptor α is expressed on cells in the subgranular zone of the dentate gyrus, and in cells with extended processes in the granular cell layer that are reminiscent of migrating neural stem cells (**A**, 10× original magnification; **B**, 40× original magnification). **C-E **Hippocampal adult neural stem cells in culture also express the GM-CSF receptor α. The expression of the GM-CSF α receptor (**C**) colocalizes with the expression of the stem cell marker nestin (**D**) on an adult neural stem cell. **E**, merged image (all size bars 50 μm). **F**, RT-PCR demonstrates presence of the GM-CSF receptor on naive NSCs from the hippocampus (HC) and subventricular zone (SVZ) in vitro.

The GM-CSF receptor is expressed in a broad variety of brain regions, with a preferentially neuronal expression pattern [3]. We also detected expression in regions where neural stem cells persist in the adult brain, such as the dentate gyrus. GM-CSFR α was expressed by cells in the subgranular zone, and in cells of the granular layer that are reminiscent of migrating stem cells (Figure 1A, B). We also detected expression of the receptor on adult neural stem cells from the hippocampus, that also stained positive for the stem cell marker nestin (Figure 1C–E). Stem cells in culture also expressed the GM-CSF receptor as judged by RT-PCR (Figure 1F).

### GM-CSF promotes neuronal differentiation of adult neural stem cells

**Figure 2:**
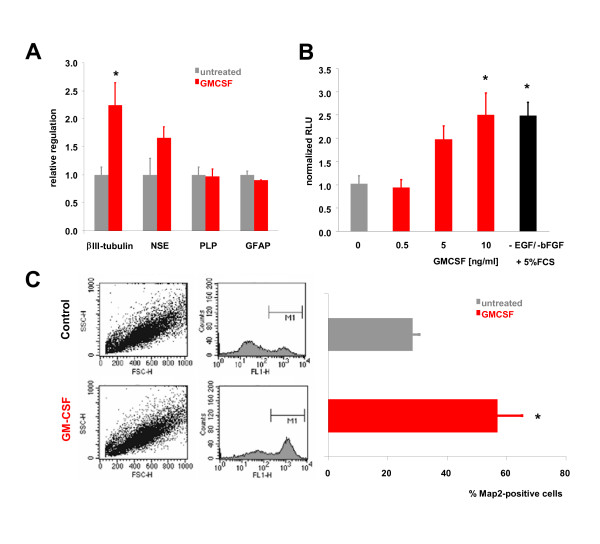
**GM-CSF induces neuronal differentiation of adult neural stem cells**. **A**, Expression of neural markers in stem cells. 3d after GM-CSF treatment of adult neural stem cells, the upregulation of the neuronal markers β III-tubulin and NSE (neuron specific enolase) was measured by qPCR. Note that there is significant induction of β III-tubulin (*, p<0.05, two-tailed t-test) whereas regulation of PLP or GFAP was not detectable. **B**, GM-CSF drives concentration-dependent neuronal differentiation of NSCs. For the luciferase reporter assay, adult neural stem cells were transfected with the pGL3-p-βIII-tubulin reporter vector and stimulated with increasing concentrations of GM-CSF. 48 h after stimulation, a concentration-dependent activation of the βIII-tubulin promoter was detected. As positive control, NSCs were treated with the standard differentiation protocol involving withdrawal of EGF and bFGF, and addition of FCS (*, p<0.05 by ANOVA and Newman-Keuls post-hoc test). **C**, Analysis of neuronal differentiation on the cellular level. FACS analysis demonstrating stem cells positive for the neuronal marker MAP-2 after treatment with GM-CSF. The percentage of MAP-2-positive cells is doubled under GM-CSF treatment (*, p<0.05, two-tailed t-test). All data are shown as mean ± SEM.

We therefore asked whether GM-CSF influenced the differentiation of adult neural stem cells. To determine if GM-CSF potentially influenced the generation of mature neural cell types we assayed the expression of the neuronal marker genes β-III-tubulin and neuron-specific enolase (NSE), the oligodendrocytic marker proteolipid protein (PLP), and the astrocytic marker glial fibrillary acidic protein (GFAP) by quantitative PCR (qPCR) in adult neural stem cells (NSCs). 3 days after GM-CSF treatment (10 ng/ml) of adult neural stem cells we detected a significant induction of β III-tubulin, and a non-significant elevation of NSE, whereas regulation of PLP or GFAP was not detectable (Figure 2A).

To determine whether this induction of mature neuronal marker expression was concentration-dependent, we assayed GM-CSF-induced differentiation by a luciferase reporter assay [6]. Adult neural stem cells were transfected with the pGL3-p-βIII-tubulin reporter vector and stimulated with increasing concentrations of GM-CSF (0.5 – 10 ng/ml). We detected a concentration-dependent activation of the β III-tubulin promoter after 48 h of GM-CSF exposure (Figure 2B). As positive control, NSCs were treated with the standard differentiation protocol involving withdrawal of EGF and bFGF, and addition of FCS. 10 ng/ml GM-CSF had the same induction potential compared to this standard protocol.

While the above assays measure marker induction on the cell population level, we used fluorescence-activated cell counting to determine differentiation on the cellular level. The percentage of MAP-2-positive cells doubled under GM-CSF treatment (Figure 2C). In summary, GM-CSF promoted neuronal differentiation of neural stem cells in vitro.

## Discussion

Recently we uncovered that the hematopoietic factor GM-CSF is also a neuronal growth factor with strong anti-apoptotic actions on neurons [3]. This characteristic is shared with two other important hematopoietic factors, EPO and G-CSF. On top of these anti-apoptotic properties, both hematopoietic factors also display neurogenic activities. EPO enhances neuronal differentiation of neural stem cells in vitro [7,8], EPO-receptor deficient mice display reduced embryonic neurogenesis [7,9], mice with a brain-specific knock-down of the EPO receptor show a decrease in stroke-induced neurogenesis and migration [9], and exogenously added EPO enhances neurogenesis and behavioural outcome after injury models in vivo [10,11]. Likewise, the G-CSF receptor is expressed on neural stem cells in the brain and in vitro [6], G-CSF stimulates neuronal differentiation of neural stem cells in vitro [6], and treatment of animal injury models with G-CSF enhances neurogenesis and functional recovery [6,12-14]. Positive effects of G-CSF on neurogenesis in an animal model for Alzheimer's disease have also been described [15]. The multimodal efficacy of these factors, combined with their established safety in other indications, has led to a number of clinical trials in stroke patients [16-19]. GM-CSF is a likewise attractive candidate for clinical development, because it is clinically in use for a number of years in hematological indications, is anti-apoptotic and neuroprotective in vivo, passes the intact blood-brain barrier [3], and also has a neurogenic potential, likely beneficial for long-term stroke recovery. Further studies need to address issues of functional recovery and neurogenesis in rodent stroke models.

Presence of the GM-CSF receptor has also been identified on neuroepithelial cells from embryonic mouse brains [20]. Addition of GM-CSF at concentrations of 0.05 to 5 μg/ml for 48 h inhibited staurosporine-induced apoptosis, and increased the number and diameter of stem cell colonies. With addition of 0.5 μg/ml GM-CSF for 48 h the authors report a diminished number of MAP-2 and GFAP-positive cells, indicating a potential negative impact of GM-CSF on (neuronal) differentiation. In general these data from the embryo support our findings on adult neural stem cells, with regard to presence of the receptor on neural stem cells, and anti-apoptotic actions of GM-CSF [3]. However, there is an apparent discrepancy in the role of GM-CSF in differentiation. While it is possible that there is a principle switch in the function of GM-CSF from a more proliferation-enhancing growth factor at the embryonic stage to a differentiation-enhancing factor in adult life, there are a number of important differences between both studies that make final conclusions difficult. The most prominent relate to the concentrations of GM-CSF employed by Kim et al., that appear unphysiologically high for a growth factor (500 ng/ml by Kim et al. vs. 10 ng/ml used in the above experiments). At least for EPO, similar functions on embryonic and adult stem cells have been described [8].

## Conclusion

Here we have shown that the GM-CSF receptor is expressed on adult neural stem cells, and that GM-CSF induces dose-dependent neuronal differentiation of these cells. This property places GM-CSF together with other hematopoietic factors that have recently been shown to function also as growth factors in the brain. On hematopoietic stem cells, GM-CSF induces the generation and maturation of neutrophilic granulocytes and monocytes/macrophages. A similar role of GM-CSF in hematopoietic and neural differentiation underlines the similarities that exist between the two stem cell populations [21,22]. It appears that the hematopoietic factors EPO, G-CSF, and GM-CSF have all evolved to serve basic cellular functions such as anti-apoptosis and differentiation in two different body compartments.

## Methods

### Cultivation of adult neural stem cells (NSCs)

Neural stem cells were obtained from the hippocampus or subventricular zone of 4–6 week old male Wistar rats as described [23]. Briefly, animals were sacrificed, brains dissected, washed in ice-cold Dulbecco's Phosphate Buffered Saline (DPBS) containing 4.5 g/L glucose (DPBS/Glc). The hippocampus and subventricular zone were dissected from 6 animals, washed in 10 ml DPBS/Glc, and centrifuged for 5 min at 1600 g at 4°C. Tissue was minced by scissors. Tissue pieces were washed again, centrifuged for 5 min at 800 g, and the pellet resuspended in 0.01 % (w/v) papain, 0.1 % (w/v) Dispase II (neutral protease), 0.01 % (w/v) DNase I, and 12.4 mM manganese sulfate in Hank's Balanced Salt Solution (HBSS). Tissue was incubated for 40 min at room temperature.

Subsequently, the suspension was centrifuged at 4°C for 5 min at 800 g, and the pellet washed three times in 10 ml DMEM-Ham's F-12 Medium containing 2 mM L-Glutamin, 100 units/ml penicillin/streptomycin. Cells were then resuspended in 1 ml neurobasalmedium containing B27 (Invitrogen), 2 mM L-glutamin, 100 units/ml penicillin/streptomycin, 20 ng/ml EGF, 20 ng/ml FGF-2, and 2 μg/ml heparin. Cells were seeded into 6-well plates at a concentration of 25,000–100,000 cells/ml, and incubated at 37°C in 5 % CO_2_. Two thirds of the medium volume was changed weekly [23].

### Immunocytochemistry

Neural stem cells were dissociated and plated on poly-L-ornithin/laminin-coated 96-well plates at a density of 30 000 cells/well. After 2 days stem cells were washed with PBS (Gibco) (37°C) and fixed with 4% paraformaldehyde for 10 min on ice. Then, cells were washed with PBS (4°C) and stored at 4°C. Cells were incubated for 10 min in 50 mM glycine in PBS, and then washed with PBS. After permeabilisation on ice using 0.2% TritonX-100 (Sigma) in PBS, cells were incubated with blocking solution (1% BSA in PBS) at room temperature. The GM-CSF receptor alpha antiserum (1:100, Santa Cruz) and the nestin antiserum (1:100, BD Transduction Laboratories) were incubated over night at 4°C. Cells were then washed with 0.1% BSA in PBS, and incubated for 1 h with the secondary antibodies (anti-rabbit-FITC and anti-mouse TRITC, 1:200; dianova) at room temperature. Cells were then washed briefly in 0.1% BSA in PBS, and stained with Hoechst 33342 (Molecular Probes) (1:10000 in PBS).

### PCR on GM-CSFRalpha

RNA of neurospheres of the hippocampus or SVZ was isolated using the Qiagen RNeasy mini kit following the manufacturers recommendations. cDNA was synthesized from 5 μg total RNA using oligodT primers, superscript II reverse transcriptase (Gibco) using standard conditions. PCR was performed for the GM-CSFR alpha chain (BR4-4s96: ACG TCG TTG GCT CAG TTA TGT C; BR4-4as272: ATTTATGTCAGAGATGGAGGATGG, product length 176 bp) using 60°C annealing and 32 cycles. Products were visualized by agarose gel electrophoresis, and ethidium bromide staining.

### Quantitative PCR

Neurospheres derived from the subventricular zone (SVZ) were stimulated with 10 ng/ml recombinant human GM-CSF (Leukine^®^, Immunex/Schering)(n = 3). 3 days after addition of GM-CSF, cells were harvested for the RNA-preparation, whereas untreated cells (n = 3) served as control. RNA of the GM-CSF-treated and untreated neurospheres of the SVZ was isolated using the Qiagen RNeasy mini kit following the manufacturers recommendations. cDNA was synthesized from 5 μg total RNA using oligodT primers, superscript II reverse transcriptase (Gibco) using standard conditions. Quantitative PCR was performed using the Lightcycler system (Roche Diagnostics, Mannheim, Germany) with SYBR-green staining of DNA doublestrands. Cycling conditions were as follows: beta III-tubulin: 3 min 95°C, 5 sec 95°C, 10 sec 65°C, 30 sec 72°C; 10 sec 87°C for 50 cycles; NSE (neuron specific enolase): 3 min 95°C, 5 sec 95°C, 10 sec 58°C, 30 sec 72°C; 10 sec 81°C for 50 cycles; PLP: 3 min 95°C, 5 sec 95°C, 10 sec 62°C, 30 sec 72°C; 10 sec 84°C for 50 cycles; GFAP: 3 min 95°C, 5 sec 95°C, 10 sec 60°C, 30 sec 72°C; 10 sec 81°C for 50 cycles. Melting curves were determined using the following parameters: 95°C cooling to 50°C; ramping to 99°C at 0.2°C/sec. The following primer pairs were used: "rat beta III-tub-716s" CCA CCT ACG GGG ACC TCA ACC AC, "rat beta III-tub-1022as" GAC ATG CGC CCA CGG AAG ACG, "rat NSE-plus" GGC AAG GAT GCC ACT AAT GT, "rat NSE -minus" AGG GTC AGC AGG AGAC TTG A, "rat PLP-518s" TCA TTC TTT GGA GCG GGT GTG, "rat PLP-927as" TAA GGA CGG CAA AGT TGT AAG TGG, "rat GFAP3'-1123s" CCT TTC TTA TGC ATG TAC GGA G, "rat GFAP3'-1245as" GTA CAC TAA TAC GAA GGC ACT C. The Lightcycler PCR was performed using the SYBR green master mix, following the manufacturers recommendations (Roche diagnostics). Specificity of product was ensured by melting point analysis and agarose gel electrophoresis. cDNA content of samples was normalized to the expression level of Cyclophilin (primers: "cyc5" ACC CCA CCG TGT TCT TCG AC, "acyc300" CAT TTG CCA TGG ACA AGA TG). Relative regulation levels were derived after normalization to cyclophilin, and comparison to the untreated cells.

### Luciferase assay

To generate the pGL3-p-βIII-tubulin experimental vector, the class III β-tubulin gene promoter (fragment -450 - +54) [24] was inserted into the pGL3-Basic firefly luciferase reporter vector (Promega) as described before [6]. Cultivation of stem cells, DNA-transfection and luciferase assay were carried out as described before [6]. Briefly, stem cells were seeded at a density of 35 000 cells/well and were cotransfected with the pGL3-p-βIII-tubulin vector and a Renilla luciferase construct with the Lipofectamine method (Invitrogen) 24 h after plating. Following the incubation of transfected cells for 24 h, cells were stimulated with various concentrations of GM-CSF in Neurobasal medium (0.5 ng/ml, 5 ng/ml, 10 ng/ml) for 48 h. As positive control for in vitro differentiation, stem cells were treated by withdrawing mitogens and adding 5% fetal calf serum (FCS). Using the Dual-Luciferase Reporter Assay System (Promega) the ratio of luminescence signals from firefly and renilla luciferase was obtained (Berthold Technologies, Mithras LB 940).

### FACS analysis

For differentiation experiments, adult NSCs were plated in 15 cm^2 ^culture flasks at a density of 4 million cells and were treated once with 10 ng/ml GM-CSF (n = 3). A single cell suspension was made by triturating the neurospheres in 1 ml plastic pipettes, and then collected by centrifugation. After resuspension in 1× phosphate-buffered saline (PBS), the cells were fixed with 1% PFA. The cells were incubated for 15 min on ice, washed once with 1× PBS and then permeabilised by resuspension in 0.2% tween20. After an incubation on ice for 15 min, fetal calf serum (FCS) was added in a 1:50 dilution for blocking. The cells were incubated for 2 h on ice with a MAP2 antibody (Sigma; 1:50) and washed three times with 0.1% tween20. Following an incubation for 30 min on ice with a donkey anti-mouse FITC-conjugated secondary antibody (Dianova), the cells were washed again three times with 0.1% tween20, and finally resuspended in 1× PBS for FACS analysis. Flow cytometry of cells was performed on a FACSCalibur (Becton-Dickinson).

## Competing interests

The author(s) declares that there are no competing interests.

## Authors' contributions

AS conceived and planned the experiments, CK, RL, CP conducted all experiments and analyzed the data, WRS helped with discussions of the data, AS and CK wrote the manuscript. All authors read and approved the final manuscript.
